# Completed genome segments of Maciel, Lechiguanas, and Laguna Negra orthohantaviruses

**DOI:** 10.1128/mra.00441-24

**Published:** 2024-10-14

**Authors:** Elizabeth Shedroff, Shannon L. M. Whitmer, Melissa Mobley, Maria Morales-Betoulle, Maria Laura Martin, Julia Brignone, Carina Sen, Yael Nazar, Joel M. Montgomery, John D. Klena

**Affiliations:** 1 Viral Special Pathogens Branch, Centers for Disease Control and Prevention, Atlanta, Georgia, USA; 2 Instituto Nacional de Enfermedades Virales Humanas Dr. Julio I. Maiztegui, Pergamino, Argentina; Portland State University, Portland, Oregon, USA

**Keywords:** *Orthohantavirus*, hantavirus, NGS, virology, taxonomy

## Abstract

New World orthohantaviruses are rodent-borne tri-segmented viruses that cause hantavirus cardiopulmonary syndrome in humans in the Americas. Molecular diagnostics for orthohantaviruses can be improved with more sequence data. Reported here are completed genomes for Lechiguanas, Maciel, and Laguna Negra viruses.

## ANNOUNCEMENT

Orthohantaviruses (*Hantaviridae* family*, Mammantavirinae* subfamily) are rodent-borne tri-segmented viruses that cause human disease (1, 2). The small (S) segment encodes the nucleocapsid, the medium (M) segment encodes the glycoprotein, and the large (L) segment encodes the viral polymerase. New World (NW) hantaviruses in the Americas cause hantavirus cardiopulmonary syndrome in humans and diverse viral strains circulate across the continent. Sequencing additional NW hantaviruses improves the breadth of molecular diagnostics assays. Additionally, the International Committee on Taxonomy of Viruses (ICTV) is reorganizing orthohantaviruses using complete genomes; incomplete genomes place several viruses at risk of possible declassification (3). Currently, full-length L segments are missing for Laguna Negra (LNV), Lechiguanas (LECV), and Maciel (MACV) viruses and a complete M segment is unavailable for MACV (4–8). Reported here are an LECV virus S segment, MACV M segment, and LECV, MACV, and LNV virus L segments. Sequences originated from viral isolates previously generated from rodent specimens collected in Argentina and Paraguay (4, 7).

Viral isolates from freezer stocks were cultured in Vero E6 cell lines, and RNA was extracted using the Tripure method described by Whitmer et al. (9). cDNA library preparation was performed using the NEBNExt Ultra II RNA Directional Library Preparation Kit (NEB) and sequenced with an Illumina MiSeq (300 cycles V2 kit) or MiniSeq (High Output 2 × 150 cycles) (9). Reads were trimmed for quality [prinseq-lite -min_qual_mean 25 -trim_qual_right 20 -min_len 50 (10)], and guided *de novo* assembly was performed by iteratively mapping contigs [spades.py -k auto (11)] and trimmed reads to reference sequences [LNV – JX443696 LANV-2 L segment, MACV – MN258160 and NC_003468 Andes virus (ANDV) L, and AF028027 MACV M; LECV – MN258160 ANDV L and NC_003468 ANDV L] with Geneious mapper set to high sensitivity/slow (Geneious Prime 2022.0.2, https://www.geneious.com). All bioinformatic tools were run with default parameters unless otherwise specified. The total number of reads, average read length, and fold coverage for each viral segment are described in Table 1.

**TABLE 1:** Summary of Hantavirus sequencing and coverage depths

Virus, isolate	Accession numbers	Total reads	Average length (bp)	L segment coverage depth	L segment %GC content	M segment coverage depth	M segment %GC content	S segment coverage depth	S segment %GC content
Maciel	PP058739, PP058741	14,663,556	121	4,056×	37.0	9,395×	40.5	^*a*^	
Lechiguanas, 15631	PP058737, PP058740	10,811,950	117	2,656×	37.9	^*a*^		1,287×	40.8
Laguna Negra, 807266	PP058738	1,831,368	150	12,519×	36.7	^*a*^		^*a*^	

^*a*^ Viral segment already available on GenBank.

**Fig 1:**
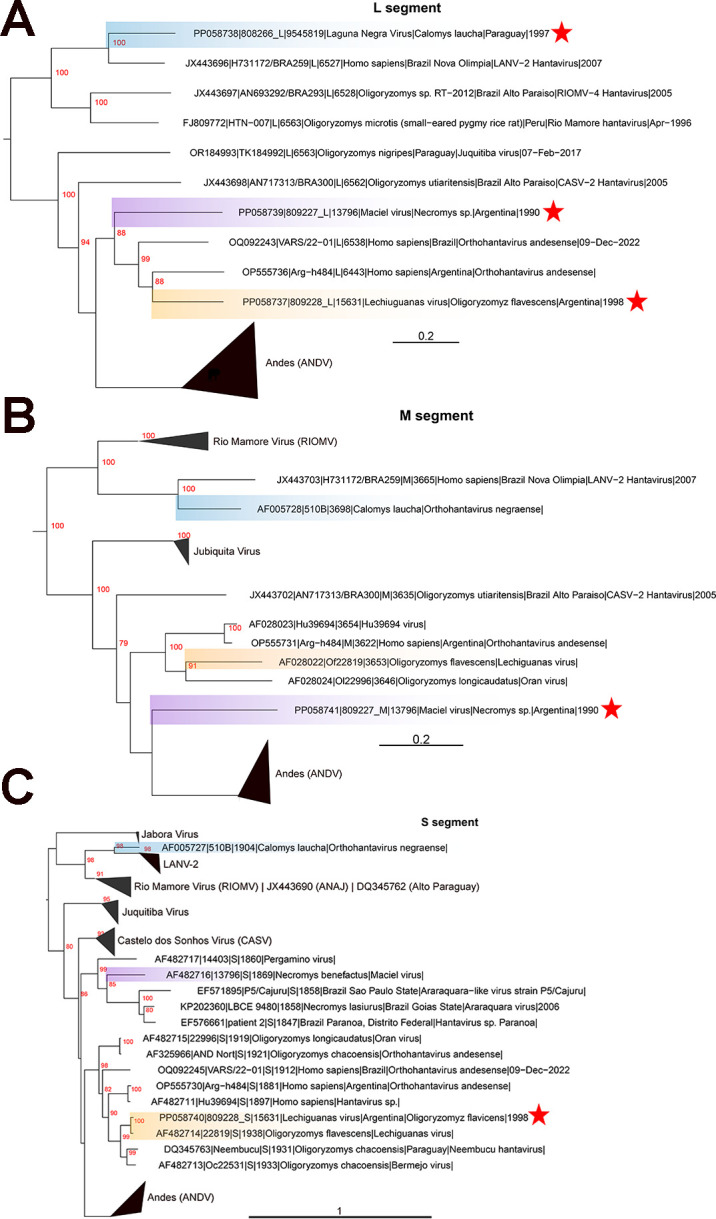
Inferred phylogenetic relationships of coding complete South American orthohantaviruses. Nucleotide alignments were generated with MAFFT (version 7.471) using default settings for full-length L segments (A), M segments (B), and S segments (C), and trees were generated by maximum likelihood using RAxML (version 7.3.0, -m GTRGAMMA -p $RANDOM -f a -x $RANDOM -N 1000). Major clades are collapsed and labeled. Trees are midpoint rooted with bootstrap support (*n* = 1,000 iterations) highlighted in red at each node, and scale bars are in units of substitutions per site. Laguna Negra virus is highlighted in blue, Maciel virus is highlighted in purple, and Lechiguanas virus is highlighted in orange. Red stars indicate new sequences.

Phylogenetic analysis revealed a well-supported relationship between the new LNV L segment and the LANV-2 virus L segment; this relationship also extends to the pre-existing M and S segments (Fig. 1A through C). The MACV S segment is most closely related to the Pergamino virus from Argentina and is ancestral to the Brazilian Araraquara clade (Fig. 1C). The new MACV M and L segments also exhibit a similar relationship as the S segment and are most closely related to the ANDV clade and ANDV from Argentina and Brazil, respectively (Fig. 1A and B). The LECV S segment is located in a clade containing Oran virus from Argentina, ANDV from Brazil and Argentina, Bermejo virus from Argentina, and Neembucu virus from Paraguay (Fig. 1C). The M and L segments are missing for many of these diverse hantaviruses, but the LECV M segment and new L segment still exhibit a close relationship with ANDV and Oran viruses from Argentina, respectively (Fig. 1A and B).

The addition of new and diverse sequence data will enable the improvement of sequence-based diagnostic assays so that they can detect a broader range of South American hantaviruses. Additional sequences also provide full genome data for species designation by ICTV.

## Data Availability

Raw data from this Next-Generation Sequencing project have been deposited in NCBI’s SRA under the BioProject number PRJNA1050174, BioSample accession numbers SAMN38726628, SAMN38726629, and SAMN38726630, and SRA accession numbers SRR27225766, SRR27225767, and SRR27225765. Assembled genome sequences are publicly available under GenBank accession numbers PP058737-PP058741.
